# Et tu, Brute? Not Even Intracellular Mutualistic Symbionts Escape Horizontal Gene Transfer

**DOI:** 10.3390/genes8100247

**Published:** 2017-09-29

**Authors:** Sergio López-Madrigal, Rosario Gil

**Affiliations:** 1 Biologie Fonctionnelle Insectes et Interactions, UMR203 BF2I, INRA, INSA-Lyon, Université de Lyon, 69100 Villeurbanne, France; sergio.lopez@uv.es; 2 Institute for Integrative Systems Biology (I2SysBio), Universitat de València/CSIC, 46980 Paterna (València), Spain; 3 Departament de Genètica, Universitat de València, Dr. Moliner, 50, 46100 Burjassot (València), Spain

**Keywords:** horizontal gene transfer (HGT), nutritional symbiosis, insects, intracellular bacteria, integrative evolution

## Abstract

Many insect species maintain mutualistic relationships with endosymbiotic bacteria. In contrast to their free-living relatives, horizontal gene transfer (HGT) has traditionally been considered rare in long-term endosymbionts. Nevertheless, meta-omics exploration of certain symbiotic models has unveiled an increasing number of bacteria-bacteria and bacteria-host genetic transfers. The abundance and function of transferred loci suggest that HGT might play a major role in the evolution of the corresponding consortia, enhancing their adaptive value or buffering detrimental effects derived from the reductive evolution of endosymbionts’ genomes. Here, we comprehensively review the HGT cases recorded to date in insect-bacteria mutualistic consortia, and discuss their impact on the evolutionary success of these associations.

## 1. Introduction

### 1.1. Horizontal Gene Transfer: Molecular Signatures and Mechanisms

Vertical inheritance between generations, via sexual or asexual reproduction, represents the main mechanism for the transmission of genetic material in nature [1]. Nevertheless, genetic information can also be transmitted between reproductively isolated species via Horizontal Gene Transfer (HGT). This phenomenon is governed by three major mechanisms in prokaryotes: transformation (i.e., direct uptake of exogenous DNA), conjugation (i.e., plasmid-mediated uptake of exogenous DNA), and transduction (i.e., virus-mediated uptake of exogenous DNA). More recently, genetic transfer through prophages-derived gene transfer agents (GTAs) and cell fusion have been described [2]. On the other hand, the mechanisms allowing for HGT in eukaryotes remain unclear. Evidences suggest the key involvement of transposable elements [3,4,5,6,7], bacteriophages [8], giant viruses [9], and extracellular vesicles such as exosomes [10] in bacteria-to-animals transfer of genetic material.

Mutational change and subsequent selection might lead to the appearance of novel genes after the duplication of pre-existing loci. Alternatively, genes that have already undergone selective pressures can be directly transferred between different species via HGT [11]. Despite the evident evolutionary advantages of HGT (see Section 1.2), newly acquired loci often function inefficiently within their new genomic background [12,13] and/or generate detrimental side effects [14]. HGT-associated costs are related to several non-mutually exclusive phenomena. These include genetic/genomic features disruption [15,16]; sequence-specific signatures of the horizontally acquired loci [17]; sequestration of cell limited resources due to transcription and translation of gene products encoded by the foreign DNA [18]; cytotoxic effects due to misfolded proteins [19,20]; disruption of fine-tuning of cellular networks caused by changes in protein dosage [13,21] or inefficient interaction with local proteins due to the lack of molecular co-evolution [22,23]; and system-level effects derived from direct or indirect impact of acquired loci on the regulation of transcriptional patterns or the concentration of signaling metabolites [24,25].

Due to the HGT-associated costs, the kind of genes and pathways maintained over extended timescales after being transferred are highly biased. When present, horizontally acquired sequences are detected by using probabilistic methods, including molecular phylogenetics, codon usage, and oligonucleotide composition analyses [2]. In addition, synteny-based evidences or shared ecological niches between donor and recipient species can be used to further support the HGT-hypothesis.

### 1.2. Horizontal Gene Transfer as an Evolutionary Force

Horizontal transfer of genetic material plays a major evolutionary role among prokaryotes [26,27], explaining their extensive ecological diversification [28,29,30], and being relevant for bacterial evolution at least since the origins of the bacterial divisions [31]. Eukaryotes, however, present several barriers to HGT, including the selective double membrane of the cellular nucleus, the required adjustment of acquired genetic elements to the eukaryotic transcription machinery, and the need to affect the germ cell line in order to ensure intergenerational transmission in multicellular organisms [11,32]. In spite of this, prokaryotes-to-eukaryotes HGT events have drastically influenced eukaryotes early evolution. According to the Serial Endosymbiosis Theory (SET), free-living alphaproteobacteria and cyanobacteria were the ancestors of mitochondria and chloroplast, respectively. Their functional integration with early eukaryotes led to the organelles drastic genome reduction and horizontal transfer of both RNA- and protein-coding genes to the eukaryotic nuclear genome [1,33,34]. This phenomenon apparently keeps playing an important role in eukaryotes adaptive evolution [35]. Thus, many HGT events detected in fungi [36,37,38], plants [9,39], and animal genomes (see next sections for further details) involve bacteria as donor species. In general, bacteria possessing the ability to transfer DNA to eukaryotes closely interact with eukaryotic hosts (i.e., they maintain symbiotic associations of parasitic or mutualistic nature), and show high levels of genome plasticity and gene motility by means of a relevant mobilome. In addition, certain bacterial structures such as Type IV Secretion System (T4SS), the only natural bacterium-to-eukaryote DNA transfer system known so far [40], might facilitate this kind of transfer.

Next-generation sequencing technologies are yielding a growing body of evidence on HGT signatures in animal genomes [41]. Most HGT events affect invertebrates that display close associations with a broad range of microorganisms [42,43], and whose simpler structural organization is expected to increase the accessibility of their germline to exogenous DNA. In some cases, HGT drastically impacts animals’ biology. For instance, the human parasitic nematode *Brugia malayi* encodes an essential ferrochelatase gene of prokaryotic origin [44], and the transfer of a nearly complete *Wolbachia pipientis* genome has triggered the evolution of a new sex chromosome in pill bugs [45]. Furthermore, HGT have apparently allowed for the colonization of novel ecological niches. Thus, a number of genes encoding plant cell-wall degrading enzymes such as cellulases, xylanases, pectate lyases, and polygalacturonases have been found in plant–parasitic nematodes [4,46]. In the same line, the adaptation to the herbivorous lifestyle of the coffee berry borer beetle *Hypothenemus hampei* and the mustard leaf beetle *Phaedon cochleariae* has been facilitated by horizontally acquired genes (i.e., mannanase and xylanase, respectively), likely coming from the gut microbiota [3,6]. In some cases, selective advantages supplied by horizontally transferred loci might not be so obvious. For example, the genetic diversity of bdelloid rotifers provided by massive HGT from bacteria, fungi and plants might both compensate for their unisexual reproductive strategy and enable tolerance against desiccation [47].

## 2. Insects-Bacteria Endosymbioses

**Figure 1:**
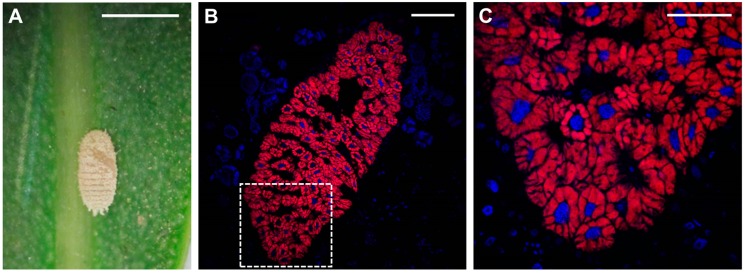
*Phenacoccus peruvianus*/”*Candidatus* Tremblaya phenacola” symbiotic system. (**A**) Early nymph of *P. peruvianus*; (**B**,**C**) Confocal images showing a complete bacteriome section (**B**) and the magnification of the area within the dashed square (**C**). DAPI (4′,6-diamidino-2-phenylindole)-stained nuclei appear in blue; EUB338-probed bacteria appear in red. Fluorescence in Situ Hybridization (FISH) procedure is described in [55]. Scale bars: 1 mm (**A**), 100 μm (**B**), 50 μm (**C**).

Nutritional symbioses between insects and intracellular bacteria are among the closest inter-domain associations found in nature. This kind of symbiosis mostly affects insects from orders Blattaria, Curculionidae and Hemiptera [48], being considered a key factor for their evolutionary success. Obligate mutualistic bacteria, also known as primary endosymbionts (P-endosymbionts), inhabit insect polyploid cells (bacteriocytes) which usually aggregate into a specialized bacteria-bearing organ (bacteriome; Figure 1). Many of them complement their host’s unbalanced diets (e.g., plant sap, vertebrates blood), allowing insects to colonize nutritionally poor niches. In coherence, a link between the diet composition and the nutritional role of the bacterial endosymbiont is usually observed, no matter the lifestyle or genetic repertoire of its free-living ancestor [49,50]. For example, phloem sap is enriched in carbohydrates but contains very small amounts of lipids, proteins or vitamins [51,52,53]. While most lipids can be synthesized from carbohydrates, proteins and vitamins cannot, due to the scarcity of nitrogenous precursors. Thus, provision of essential amino acids and vitamins is usually the nutritional role of P-endosymbionts in sap-feeding insects [54].

P-endosymbionts are essential for their host survival and reproduction, being fixed (i.e., 100% prevalence) in the host population [56]. Moreover, they are vertically transmitted from mothers to offspring [57], so that hosts and long-term P-endosymbionts show congruent phylogenies due to coevolution [58,59,60,61]. Both the intracellular environment they inhabit and the population dynamics associated to vertical inheritance drive the dramatic genome shrinkage, also known as genomic reduction syndrome, undergone by these bacteria during the transition to the intracellular lifestyle [43]. Bacteriocyte cytoplasm represents a rich, stable niche, rendering unnecessary or redundant many of the genes encoded by free-living bacteria genomes. Furthermore, periodical bottlenecking associated to the vertical transmission of P-endosymbionts between host generations increases the evolutionary impact of genetic drift [62,63], so that slightly deleterious mutations accumulate in loci under relaxed purifying selection, which then get inactivated and subsequently eliminated. Genes involved in DNA uptake, repair, and recombination are early affected by this genome-reduction process, usually leading to small, AT-enriched, structurally stable genomes showing few (if any) pseudogenes or mobile elements [57]. This is a progressive phenomenon yielding highly simplified genomes (i.e., the tiny genomes exhibited by symbionelles [64]). Highly reduced genomes lack essential functions either for the fulfillment of symbiosis requirements or for the maintenance of P-endosymbionts themselves [65,66,67,68]. In these cases, it has been hypothesized that the role of missing genes is taken over by the cooptation/functional plasticity of remaining loci [69], the recruitment of newly acquired symbionts (i.e., metabolic complementation and/or replacement by a co-primary endosymbiont; [70,71]), or the participation of the host via eukaryotic or horizontally-acquired prokaryotic genes [72].

In addition to nutritional symbioses, transient associations between insects and facultative/secondary symbionts (S-symbionts) have been described. In contrast to obligatory endosymbionts, S-symbionts display only partial infection of host populations. Moreover, they can be laterally transferred between host species [73,74], and are found within cell types other than bacteriocytes, as well as free in the host haemolymph [75,76,77]. Some of them, collectively known as reproductive parasites, are able to distort their host’s sex ratio by inducing reproductive alterations such as cytoplasmic incompatibility, parthenogenesis induction in haplo-diploid species, feminization of genetic males, or male-killing [78,79]. Other S-symbionts are known to enrich the genotype of insects by providing them with adaptive ecological traits. For instance, some S-symbionts broad the food plant range of their host or enhance their resistance to both biological and/or physical environmental stress [80].

## 3. Horizontal Gene Transfer in Insects-Bacteria Endosymbiotic Systems

### 3.1. Bacteria to Insects and Vice Versa

*Wolbachia* is among the most widespread intracellular bacteria described [79]. A variety of HGT events between *Wolbachia* and many of its insect hosts have been noticed, in coherence with its high estimated prevalence (from 20% to 66% insect species; [81,82]), and its close association with insects’ germ line [79]. These include both the *Wolbachia*-to-host transfer of genetic material [8,83,84] and vice versa [85]. Nevertheless, the evolutionary significance of these HGT events is hard to demonstrate [86], since most horizontally acquired genes are transcriptionally inactive and/or exhibit premature stop codons, frameshifts, or retroelement insertions [87].

**Figure 2:**
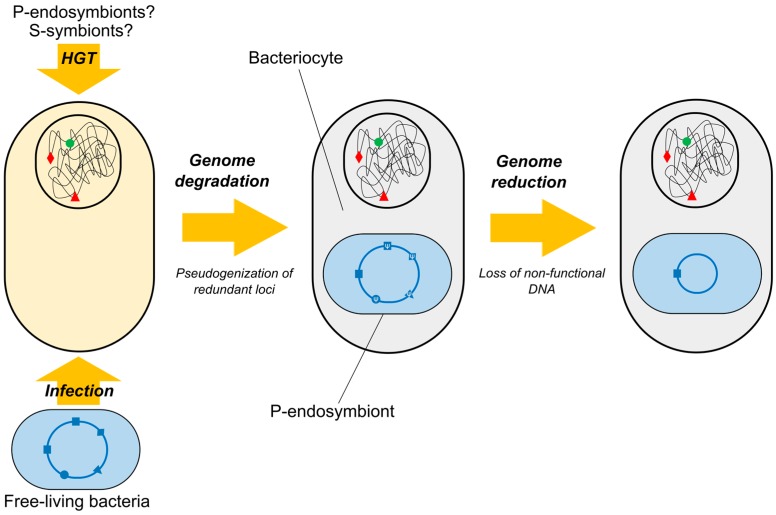
Reductive evolution of P-endosymbionts in insects whose genomes carry loci of prokaryotic origin. Horizontally acquired genes from ancient P-endosymbionts/S-symbionts (green/red symbols), as well as ongoing reductive evolution of current P-endosymbiont through inactivation and loss of redundant loci (square, triangle, rhombus, circle) are shown. HGT: Horizontal Gene Transfer.

In contrast to this general view, HGT is considered one of the signatures of genome coevolution in insect-bacteria nutritional endosymbioses [72]. As demonstrated by the recent accumulation of hologenomes (i.e., coupling of both endosymbiont and host genomic information), the horizontal acquisition of prokaryotic genes by insects maintaining nutritional symbiosis with intracellular bacteria has allowed for further integration of the corresponding consortia (Figure 2). Examples have been recently described in the pea aphid *Acyrthosiphon pisum*, the citrus mealybug *Planococcus citri*, the hackberry petiole gall psyllid *Pachypsylla venusta*, and the cotton whitefly *Bemisia tabaci*, which are representatives of all insect families within the suborder Sternorrhyncha [88,89,90,91,92]. Despite considerable overlap in the involved functions, most of the homologous loci acquired by mealybugs, psyllids and whiteflies clearly derive from independent transfer events, since they are not phylogenetically related (Table 1). Strikingly, most of the acquired loci appear to be transferred from transient S-symbionts and/or former, already replaced, P-endosymbionts. Taking into account the fixation of P-endosymbionts in the host populations, as well as their vertical transmission via eggs colonization, this probably indicates that (1) bacteria-to-insect HGT are rare molecular events; and/or (2) P-endosymbionts are bad candidates for successful DNA emission, likely because of the mutational bias and the low protein stability derived from their genomic reductive syndrome [93,94].

Horizontally acquired genes mediating insect–bacteria nutritional symbiosis were first described in the pea aphid *A. pisum* (Hemiptera: Aphididae), which maintains a long-term mutualistic association with the gammaproteobacterial endosymbiont *Buchnera aphidicola* [95]. A total of 12 prokaryotic genes or gene fragments have been found in the *A. pisum* genome, only two of them (the pseudogenes ψ*dna*E and ψ*atp*H) being likely acquired from its P-endosymbiont *B. aphidicola* [88,89]. The other 10 genes of prokaryotic origin present in the host’s nuclear genome encode enzymes involved in peptidoglycan metabolism: several LD-carboxypeptidases (LdcA, LdcA1 and LdcA2; EC:3.4.17.13) and an *N*-acetylmuramoyl-l-alanine amidase (AmiD, EC:3.5.1.28), required for murein recycling [96], as well as lysozyme (bLys, EC:3.2.1.17), which hydrolyzes it [97]. In contrast to LdcA1 and AmiD, the expression of bLys was more abundant in other cell types than in the bacteriocyte, suggesting that it might be involved both in the regulation of symbiosis and the protection of *A. pisum* against the infection by exogenous bacteria [97,98]. Phylogenetic analyses showed that LdcAs, AmiD, and bLys have been horizontally acquired from rickettsial bacteria, which include many lineages (e.g., *Rickettsia*, *Wolbachia*) commonly found as S-symbionts in aphids [99,100]. In addition, five copies of the gene encoding RlpA (rare lipoprotein A) were detected. The bacterial source and function of this gene is still unclear. Nevertheless, protein localization of RlpA4 showed that it is targeted to *B. aphidicola* cells, which demonstrate that RlpA is both functional and tightly involved in maintenance/control of the nutritional symbiosis between *A. pisum* and its P-endosymbiont [101].

Mealybugs (Hemiptera: Pseudococcidae) maintain a variety of nutritional symbioses involving a betaproteobacterium of the genus “*Candidatus* Tremblaya” (except if replaced by a newly acquired endosymbiont [102]). While “*Candidatus* Tremblaya phenacola” remains alone in the bacteriocytes of phenacoccinae mealybugs, “*Candidatus* Tremblaya princeps” have independently engulfed several lineages of gammaproteobacteria that inhabit “*Ca*. Tremblaya princeps” cytoplasm as co-primary endosymbionts [103,104,105]. These nutritional symbioses are based on the biosynthesis of essential amino acids and vitamins, as first demonstrated by the genomic characterization of the tripartite consortium involving the citrus mealybug *P. citri* and its two co-primary endosymbionts “*Ca*. Tremblaya princeps” and “*Candidatus* Moranella endobia” [66,67]. Further exploration of the *P. citri*’s nuclear genome revealed the presence of 22 functional prokaryotic genes coming from Alphaproteobacteria, Gammaproteobacteria and Bacteroidetes, closely related to many of the most frequent insects S-symbionts (*Arsenophonus*, *Cardinium*, *Serratia*, *Sodalis*, *Rickettsia* and *Wolbachia*) [90]. Three of them (*dap*F, *lys*A and *cy*sK) apparently fulfill missing gaps in the bacterial lysine and methionine biosynthetic pathways. Another five appear to complement “*Ca*. Moranella endobia” incomplete pathways for the biosynthesis of riboflavin (*rib*A and *rib*D) and biotin (*bio*A, *bio*B and *bio*D). As previously noticed in *A. pisum*, bacterial genes involved in peptidoglycan metabolism have also been acquired by *P. citri*. These include loci devoted to peptidoglycan biosynthesis (*mur*A, *mur*B, *mur*C, *mur*D, *mur*E, *mur*F and *ddl*B) and recycling (*mlt*B and *ami*D). It has been proposed that these genes might mediate the molecular crosstalk between “*Ca*. Tremblaya princeps” and its nested endosymbiont “*Ca*. Moranella endobia“ [90,106].

Ten bacterial genes have been identified in the nuclear genome of *P. venusta*, most of them being also present in the nuclear genome of the Asian citrus psyllid *Diaphorina citri* and the potato psyllid *Bactericera cockerelli* [91]. As expected for psyllids (Hemiptera: Sternorrhyncha), *P. venusta* maintains a long-term nutritional symbiosis with the gammaproteobacterium “*Candidatus* Carsonella ruddii” [107]. In line with the findings in *P. citri*, some of the horizontally acquired genes are involved in the biosynthesis of arginine, phenylalanine and riboflavin. While the gene coding for chorismate mutase (CM, EC:5.4.99.5) appears to compensate for “*Ca*. Carsonella ruddi” functional simplification during genome shrinkage, the nuclear gene of prokaryotic origin *rib*C (encoding riboflavin synthase, EC:2.5.1.9) is the single locus devoted to riboflavin biosynthesis in the holobiont genome. Authors suggest that *rib*C might represent a vestige of an ancient complementarity for riboflavin biosynthesis with a former, already lost endosymbiont in the *Pachyspylla* lineage. In coherence, this gene is present in the genome of *D. citri*, which still requires the bacterial supply of riboflavin, according to the functional analysis of its endosymbiotic consortium [108]. On the other hand, “*Ca*. Carsonella ruddii” still retains a functional copy of *arg*H (encoding argininosuccinate lyase, EC:4.3.2.1) that is closely related to the two copies of such gene found in the host genome, suggesting that this P-endosymbiont was the bacterial donor. For the rest of horizontally acquired loci, frequent S-symbiotic bacteria such as *Rickettsia* and *Wolbachia* might be the source [91].

Finally, horizontally acquired genes were also found in the nuclear genome of the whitefly *Bemisia tabaci* (Hemiptera: Aleyrodoidea), which maintains a long-term obligatory symbiosis with the gammaproteobacterium “Ca. Portiera aleyrodidarum” [60]. In *Portiera*-BT genomes, many genes involved in the biosynthesis of essential amino acids have been inactivated (*arg*H, *dap*B) or lost (*arg*A, *arg*B, *arg*C, *arg*E, *dap*F, *lys*A, *his*D), affecting its capacity to supply arginine, lysine and histidine to its host [109,110,111]. Furthermore, “Ca. Portiera aleyrodidarum” is unable to synthesize a set of cofactors (i.e., thiamine, nicotinamide, pyridoxal-5-phosphate, folic acid, FMN/FADH, ubiquinone, heme) predicted to be required for its metabolic functions. Part of these deficiencies can be compensated by a second endosymbiont, the gammaproteobacterium “*Candidatus* Hamiltonella defensa”, which retains *dap*B, *dap*F and *lys*A (lysine biosynthesis) and encodes the complete biosynthetic pathways for six of the eight required cofactors. Nevertheless, it still lacks the complete thiamine biosynthetic pathway as well as *pho*AB, involved in folate biosynthesis. Their host, *B. tabaci*, encodes ten metabolic genes of bacterial origin [92]. Six of them are involved in the biosynthesis of the essential amino acids arginine (*arg*G and *arg*H), lysine (*dap*B, *dap*F and *lys*A), and phenylalanine (CM). The rest of the horizontally acquired genes code for proteins involved in the biosynthesis of thiamine (*bio*A and *bio*B) and the degradation of urea (DUR1,2 and AH, encoding urea carboxylase/allophanate hydrolase, EC:6.3.4.6/EC:3.5.1.54, and allophanate hydrolase, EC:3.5.1.54, respectively). The acquisition of CM, *bio*A and *bio*B, at least, is relatively ancient, since the corresponding transcripts were detected also in the greenhouse whitefly *Trialeurodes vaporariorum*. Even if “*Ca*. Portiera aleyrodidarum” and/or “*Ca*. Hamiltonella defensa” still retain homologs for all horizontally acquired loci but AH and DUR1,2, phylogenetic evidences rule out the possibility that they were the bacterial sources. In contrast, transferred genes cluster with different Gammaproteobacteria (including *Pantoea* and stinkbugs gut symbionts), Alphaproteobacteria (Rickettsiales), Bacteroidetes (*Niastella*) and Planctomycetes (*Isosphaera*).

### 3.2. Bacteria to Bacteria

As mentioned above, prokaryotes are highly prone to DNA exchange. The genomes of many insect endosymbiotic bacteria have been characterized in the last 17 years, but very few evidences for bacteria-to-bacteria gene transfer have been noticed, even if the transient infection by S-symbionts and/or the stable presence of multiple co-primary endosymbiotic species are frequent.

#### 3.2.1. Reasons behind Horizontal Gene Transfer Scarcity among Endosymbionts

The intracellular lifestyle, the vertical transmission and genomic features typically associated with their genome reduction syndrome (i.e., AT-enrichment and gene repertoire simplification) might explain the refractoriness of endosymbionts to HGT.

• *Intracellular lifestyle*: HGT-associated costs limit the divergence between receptor genomes and successfully transferred loci (see Section 1.1). Therefore, HGT incidence between closely related bacteria is significantly more frequent [112]. Closely related free-living bacteria get often in contact because they are likely to share the same habitat [113]. In contrast, endosymbionts are isolated from their ancestral habitat and close relatives when adapting to the intracellular lifestyle. Moreover, bacteriocytes isolates them from any environmental source of prokaryotic DNA. In fact, even if insects can frequently be co-infected with multiple bacterial lineages, these bacteria do not necessarily co-exist within the same bacteriocyte [99]. On the other hand, because of their intracellular distribution, endosymbiotic populations effective sizes are dramatically smaller than those of free-living bacteria, which is expected to hamper genome fluidity [114].

• *Vertical transmission:* Endosymbionts effective population size is greatly affected by serial bottlenecking during maternal transmission to the host’s next generation, which is expected to enhance genetic drift effects (i.e., elimination of at least part of the genomic polymorphism accumulating in the population [115]). This might hinder the inheritance of horizontally acquired functions, as well as the natural selection-driven amelioration of harmful pleiotropic effects associated with HGT [116,117].

• *Genome reductive evolution:* The dramatic gene repertoire simplification typically observed among long-term endosymbionts (e.g., scarcity of bacteriophages and plasmids; loss of mobile elements, T4SS, and homologous recombination genes) is expected to hamper the gene flow among endosymbionts and/or the insertion of exogenous DNA into their own replicons. In the same line, the AT-enrichment of their genomes might hinder the incidence of successful HGT events in several ways. First, gene transfer between bacterial lineages exhibiting highly different nucleotide content is unlikely, since integration of exogenous DNA via homologous recombination requires a ‘minimum efficient processing segment’ (MEPS) consisting of near-identical sequences of at least 25 bp at one or both ends of a donor segment [118,119,120]. Moreover, skewed nucleotide content leads to changes in codon usage (or even in the genetic code) [68,121] and protein amino acids composition [122]. Codon content is linked to gene expression by modulating translation efficiency and mRNA stability [123], while improper codon usage might favor a resources sequestration effect by stalling ribosomes during translation [17]. In addition, proteins containing rare amino acids are known to entail higher fitness costs than those using abundant ones [124].

#### 3.2.2. From Genetic Transfer to Genomic Fusion

In spite of evident barriers for exogenous DNA acquisition, HGT events have been noticed in several bacterial endosymbionts. For instance, genes for nitrogen fixation were horizontally acquired by “*Candidatus* Thiodiazotropha endoloripes”, endosymbiont of the lucinid bivalve *Loripes lucinalis* [125]. In the same line, “*Candidatus* Endomicrobium trichonymphae” strain Rs-D17, endosymbiont of the termite gut flagellate *Trichonympha agilis*, might have acquired loci encoding a bifunctional aldehyde dehydrogenase/ethanol dehydrogenase, amino acids transporters, and proteins involved in the biosynthesis of thiamine pyrophosphate [126]. Because of their distribution within the host (“*Ca*. Thiodiazotropha endoloripes”-containing bacteriocytes are located along the *L. lucinalis* gill lamellae, being highly exposed to the seawater flow), the lower structural complexity of hosts and/or the abundance of DNA sources in the niche they colonize (*T. agilis* inhabits the microbial-enriched gut of termites [127]), these bacteria seem relatively more exposed to exogenous DNA than insect-associated nutritional endosymbionts. In any case, HGT events accounting for a variable number of loci have been also noticed in bacteriome-associated endosymbionts of insects, confirming that HGT is a key source of evolutionary novelties throughout the prokaryotes. The examples described so far include *Wolbachia pipientis* str. wCle, “*Candidatus* Profftella armature” and “*Ca*. Tremblaya phenacola” (Table 2).

*Wobachia* wCle is associated with the bedbug *Cimex lectularius*, being essential for insect growth and reproduction via provisioning of biotin and riboflavin [128,130]. Genomic characterization of this *Wolbachia* strain revealed that, unlike all available insect-associated facultative *Wolbachia*, it encodes a complete operon for the biosynthesis of biotin (i.e., genes *bio*A, *bio*B, *bio*C, *bio*D, *bio*F and *bio*H), as well as a partial operon for the biosynthesis of thiamine (genes *ten*A1 and *thi*D, and the pseudogene ψ*thi*M). A similar operon structure for biotin biosynthesis has been identified in *Cardinium hertigii* cEper1, S-symbiont of the parasitoid wasp *Encarsia pergandiella* [131], the swine pathogen *Lawsonia intracellularis* [132], and a *Rickettsia* strain isolated from the tick *Ixodes scapularis* [133]. On the other hand, similar operon configuration for thiamine biosynthesis was identified in the fish pathogen *Francisella noatunensis* [134]. Based on this information, as well as on phylogenetic analyses, authors suggest that biotin-biosynthetic genes were probably horizontally acquired as a whole operon by an ancestor of *Wolbachia* wCle, either from *Cardinium* or *Rickettsia*, which frequently coinfect the same insect host [78]. Similarly, phylogenetic analyses showed that thiamine-biosynthetic loci were closely related to the homologous genes found in *F. noatunensis*, *Brachyspira hyodysenteriae*, *Legionella drancourtii*, and additional bacterial lineages, including representatives of the Gammaproteobacteria, Bacteroidetes, Spirochaetes, etc., supporting the HGT hypothesis. Moreover, nutritional and physiological experiments allowed them to postulate the horizontal acquisition of genes for biotin biosynthesis as a key step towards the transition between the facultative and the obligatory association of *Wolbachia* with its host [128].

Up to 20 genes have been horizontally acquired by “*Ca*. Profftella armature”, the co-primary endosymbiont of the psyllid *D. citri*, along with “*Ca*. Carsonella ruddi” str. DC [108]. Acquired genes, organized into two polyketide synthase biosynthetic gene clusters, synthesize a polyketide toxin named diaphorin. It is an analog of pederin, a defensive polyketide produced by a *Pseudomonas* symbiont of *Paederus* rove beetles, allowing the insect host to deter predators [135,136]. Authors suggest that prey-predator relationship might be involved in this HGT event, since *Paederus* rove beetles often feed on hemipteran insects [137].

In contrast to the above described examples, the case of the betaproteobacterium “*Ca*. Tremblaya phenacola” PPER, single P-endosymbiont of the bougainvillea mealybug *P. peruvianus*, goes far beyond conventional HGT, rather suggesting the formation of a new chimeric organism after the fusion of two complete genomes. Recent genome sequencing and analysis of “*Ca*. Tremblaya phenacola” PPER (Figure 1, [55]) revealed the presence of at least 80 gammaproteobacterial genes still showing a characteristic molecular signature in terms of GC-content and codon usage [129]. The functional distribution of genes is not random. According to the complexity hypothesis, genes coding for functions burdened with many complex interactions might display low HGT rates, since partial transfer of co-adapted structures is likely to end up in loss of function due to incompatibility [138,139]. Coherently, the components of complex molecular machineries share a common evolutionary origin in “*Ca*. Tremblaya phenacola” PPER [129]. Similarly to previously described symbiotic systems in mealybugs [66,67,90,105,140], bacterial supply of essential amino acids appears to be the basis of the nutritional association between “*Ca*. Tremblaya phenacola” PPER and *P. peruvianus*. Regarding this role, betaproteobacterial genes carry out the biosynthesis of methionine, threonine, isoleucine, leucine and valine, as well as the production of phenylalanine from chorismate. Only gammmaproteobacterial genes have been retained for the biosynthesis of histidine and cysteine, while both beta- and gammaproteobacterial genes collaborate in the biosynthesis of tryptophan. Overall, gammaproteobacterial genes represent 46% of PPER’s genome and place it into the *Sodalis*-allied clade. In contrast, its betaproteobacterial genes (including a single ribosomal operon) clearly place it within the “*Ca*. Tremblaya phenacola” clade. Thus, similarly to free-living bacteria undergoing extended HGT events, PPER is a nightmare for molecular taxonomists [141]. Taking into account the diverse organization of nutritional symbioses among mealybugs [102,103], a gammaproteobacterium apparently entered the symbiotic consortium in the lineage leading to “*Ca*. Tremblaya phenacola” PPER. Then, instead of replacing “*Ca*. Tremblaya” [102], genomic fusion and subsequent gene shuffling took place, likely involving homologous recombination genes encoded by the gammaproteobacterial donor [142,143]. Both the scarcity of functional redundancies and the taxonomic assignation of genes involved in the tryptophan biosynthetic pathway suggest that “*Ca*. Tremblaya phenacola” PPER coevolved with the gammaproteobacterial donor within *P. peruvianus* bacteriocytes for some time [129]. Whether a pseudococcinae-like nested consortium preceded the genomic fusion remains unclear.

## 4. Conclusions

Close association with microorganisms allowed animals to colonize highly specialized niches. This is the case of insects, whose facultative/obligatory association with mutualistic intracellular bacteria is considered essential for their evolutionary success. The ever-increasing availability of genomic data has highlighted the high impact of inter-domain associations on the horizontal acquisition of exogenous DNA by insects. Although most of the cases appear to represent transient transfers of genes, lacking an effective integration in the biology of the recipient species, recent findings strongly suggest that HGT might play a key role in the fine-tuning of mechanisms allowing for the maintenance and regulation of insect-bacteria nutritional symbioses. In the holobiont era, further analyses of available genomic/transcriptomic data, exploration of additional symbiotic models, and empirical assessment of the adaptive value of transferred loci are expected to enhance this new paradigm.

## Figures and Tables

**Table 1:** Bacteria-to-host genetic transfers. Horizontally acquired genes mediating insect-bacteria nutritional symbioses.

Host	Endosymbiont	Gene Number	Function	Source	Ref.
*Acyrthosiphon pisum*	*Buchnera aphidicola*	12	peptidoglycan metabolism (defensive, control)	*Rickettsia*, *Wolbachia*	[88,89]
*Planococcus citri*	“*Ca*. Tremblaya princeps” “*Ca*. Moranella endobia”	22	Lys, Met, riboflavin and biotin biosynthesis (nutritional) peptidoglycan metabolism (control)	*Arsenophonus*, *Cardinium*, *Rickettsia*, *Serratia*, *Sodalis*, *Wolbachia*	[90]
*Pachypsylla venusta*	“*Ca*. Carsonella ruddii”	10	Phe, Arg, riboflavin biosynthesis (nutritional) DNA mismatch repair (informational)	*Carsonella*, *Rickettsia*, *Wolbachia*	[91]
*Bemisia tabaci*	“*Ca*. Portiera aleyrodidarum” “*Ca*. Hamiltonella defensa”	10	Arg, Lys, Phe, thiamine biosynthesis/ urea degradation (nutritional)	*Pantoea* & stinkbugs gut symbionts, *Rickettsiales*, *Niastella*, *Isosphaera*	[92]

**Table 2:** Bacteria-to-bacteria horizontal gene transfer (HGT) events involving nutritional endosymbionts of insects.

Host	Endosymbiont	Gene Number	Function	Source	Ref.
*Diaphorina citri*	“*Ca*. Carsonella ruddii” “*Ca*. Profftella armatura”	20	diaphorin biosynthesis (defensive)	*Paederus*-associated *Pseudomonas*	[108]
*Cimex lectularius*	*Wolbachia pipientis*	9	biotin and thiamine biosynthesis (nutritional)	*Cardinium, Rickettsia*	[128]
*Phenacoccus peruvianus*	“*Ca*. Tremblaya phenacola”	80	nutritional informational	*Sodalis*-allied clade	[129]
